# Selective Effect of Physical Fatigue on Motor Imagery Accuracy

**DOI:** 10.1371/journal.pone.0047207

**Published:** 2012-10-17

**Authors:** Franck Di Rienzo, Christian Collet, Nady Hoyek, Aymeric Guillot

**Affiliations:** 1 CRIS EA 647, Performance Mentale, Motrice et du Matériel (P3M), Université Claude Bernard Lyon 1, F-69000 Villeurbanne Cedex, France; 2 Institut Universitaire de France, F-75000 Paris, France; Universidad Europea de Madrid, Spain

## Abstract

While the use of motor imagery (the mental representation of an action without overt execution) during actual training sessions is usually recommended, experimental studies examining the effect of physical fatigue on subsequent motor imagery performance are sparse and yielded divergent findings. Here, we investigated whether physical fatigue occurring during an intense sport training session affected motor imagery ability. Twelve swimmers (nine males, mean age 15.5 years) conducted a 45 min physically-fatiguing protocol where they swam from 70% to 100% of their maximal aerobic speed. We tested motor imagery ability immediately before and after fatigue state. Participants randomly imagined performing a swim turn using internal and external visual imagery. Self-reports ratings, imagery times and electrodermal responses, an index of alertness from the autonomic nervous system, were the dependent variables. Self-reports ratings indicated that participants did not encounter difficulty when performing motor imagery after fatigue. However, motor imagery times were significantly shortened during posttest compared to both pretest and actual turn times, thus indicating reduced timing accuracy. Looking at the selective effect of physical fatigue on external visual imagery did not reveal any difference before and after fatigue, whereas significantly shorter imagined times and electrodermal responses (respectively 15% and 48% decrease, p<0.001) were observed during the posttest for internal visual imagery. A significant correlation (r = 0.64; p<0.05) was observed between motor imagery vividness (estimated through imagery questionnaire) and autonomic responses during motor imagery after fatigue. These data support that unlike local muscle fatigue, physical fatigue occurring during intense sport training sessions is likely to affect motor imagery accuracy. These results might be explained by the updating of the internal representation of the motor sequence, due to temporary feedback originating from actual motor practice under fatigue. These findings provide insights to the co-dependent relationship between mental and motor processes.

## Introduction

Motor imagery (MI) is the mental simulation of an action without any associated overt movement. Neuroimaging studies strongly support the principle of functional equivalence between MI and physical practice of the same movement (PP) [1]–[3]. While MI is mediated by similar neural networks than those activated during motor preparation and execution, the neural substrates underlying these two tasks are hierarchically organized [4]. At the peripheral level, MI elicits comparable autonomic responses to those observed during PP [5], [6], while both EMG and TMS correlates of MI concur to support that the somatic motor command might indeed be programmed during MI [7], [8]. Interestingly, Schwoebel et al. [9] reported that a stroke patient with bilateral parietal brain damage lost the ability to inhibit the motor command during MI, and fully executed the actions he imagined. Central and peripheral neurophysiological correlates of MI support that it should not be artificially decoupled from the action itself, but rather be placed along a continuum extending from the overt movement to its mental representation [10]. This point is further supported by several studies stating that the ability to physically perform an action is essential for accurate MI performance of the same task [11], [12]. Congruency between MI and PP supports MI use in sports and more generally in motor performance processes, e.g. during rehabilitation of motor functions [2], [13]. Practically, MI is a relevant training technique which may improve motor skills [14]. However, while the combination of MI and PP is more efficient than PP alone, MI does usually not outperform PP [15]–[17]. Therefore, MI should be considered an efficient complement of PP, yet not a substitute *per se* [16], [18]. Furthermore, the rules for MI practice have been established at the scope of its closeness with PP. Holmes and Collins [19] suggested that since MI shares the same central processes as actual motor planning and programming, most components of PP should be reproduced during MI. This theoretical stance supports most recommendations for MI use to improve motor performance, like performing MI in an environmental context matching PP conditions [20], [21] and preserving the spatio-temporal characteristics of actual movement [22]. MI should also be performed at the same arousal level or, at least, close to that observed during PP [23]. More generally, several models were specifically designed to promote the best rules for MI practice. In their well-known PETTLEP model (Physical, Environment, Task, Timing, Learning, Emotion, and Perspective), Holmes and Collins [19] provided a detailed description of the key-components that should be considered to ascertain MI efficacy. Other researchers proposed reliable imagery frameworks covering important aspects of imagery use [24]–[26]. Likewise, Guillot and Collet [13] developed the Motor Imagery Integrative Model in Sport which reviews key MI components that need to be trained to ensure effective imagery interventions. They reported that mental fatigue might occur rapidly during mental training, and thus that MI sessions might benefit from limited successive trials [14], [27], [28].

Despite the great number of imagery training frameworks mentioned above, only few directly questioned the effects of physical fatigue on MI performance. This is somewhat intriguing as the use of MI during actual training, simultaneously with physical practice, is usually recommended [29], [30]. Muscle fatigue affects performance due to peripheral factors (including motor output conduction, substrate depletion and metabolite accumulation). Spindle excitability is also altered [31], thus leading to erroneous sensory feedback integration [32]. Likewise, muscle fatigue is likely to affect somesthetic perception and involve perturbation of the body schema [32], [33]. As MI is supported by central processes and differentially integrates the actual body state according to the type of imagery [34], the extent to which MI performance might be affected by peripheral fatigue should be questioned.

On our own knowledge, only two studies investigated the effects of muscle fatigue on MI ability. In a pioneering work, Guillot et al. [35] reported that local muscle fatigue did not alter MI accuracy, on the basis of comparable MI duration before and after fatigue. Thus, athletes were able to preserve the temporal organization of the movement during its mental representation. Accordingly, MI was probably mainly performed on the basis of central information from procedural memory, and to a lesser extent with reference to peripheral information based on the actual state of the motor system. Furthermore, self-estimation and autonomic nervous system correlates of MI vividness were not altered, hence suggesting that the ability to form mental images was preserved despite metabolite accumulation and substrate depletion. Although it was premature to draw final conclusions as only one type of fatigue was tested, Guillot et al. [35] argued that MI could be combined with motor performance during physical training sessions without detrimental effect upon actual execution. In a second study, Demougeot and Papaxanthis [36] observed that MI durations of arm pointing movements between three targets decreased after a physically-fatiguing exercise. The authors stated that temporal discrepancies might be explained by changes in the forward model for motor acts after local fatigue, likely to bias temporal motor prediction. Interestingly, as imagined times of the contralateral non-fatigued arm remained unchanged, the authors argued that changes in MI times may not be due to a general perception of fatigue.

To summarize, experimental studies examining the effect of muscle fatigue on MI are sparse and generated divergent findings. Unlike previous studies examining the effects of local muscle fatigue on MI accuracy, we aimed at investigating the effect of general fatigue occurring during intense sport sessions on MI accuracy. We hypothesized that physical fatigue from which athletes do not recover rapidly is likely to affect the individual ability to perform MI accurately. Further, this effect might be selective depending on individual MI abilities.

## Methods

### Participants

Twelve swimmers of regional level, including 9 males (mean age = 15.5, standard deviation = 0.96 years, mean years of practice = 5.27, standard deviation = 1.75) participated in the experiment. They signed an informed consent form after the study was approved by the University Institutional Review Board. Written consent was also obtained from the next of kin, carers or guardians on the behalf of the adolescents participating in the study. All were naive to regular MI use in sport, and were explained that MI consisted into mentally rehearsing an action without actually executing it. All participants were free of any recent injury, and had normal vision. The procedure was described accurately and instructions regarding the motor task and questionnaires were previously given, while no information was provided about the objectives of the study, or about the dependent variables of interest.

### Experimental Design

The experiment consisted in a preliminary session, scheduled 7 days before the experimental session. During this first step, we assessed the individual Maximal Aerobic swimming Speed (MAS) using an incremental swimming test. Participants also completed the VMIQ-2 questionnaire [37] to estimate individual MI vividness in three different imagery modalities. Firstly, participants performed and rated the vividness of 12 MI tasks using external visual imagery (EVI, i.e. MI of the self-performed action from an external viewpoint). Secondly, they completed the 12 same items using an internal visual perspective (IVI, i.e. seeing oneself performing the action at the first-person perspective). They finally completed the items using kinesthetic imagery, *i.e.* MI based on somesthetic information generated by actual practice, although these data were not considered in subsequent analyses. The processing rules of the VMIQ-2 were individually explained, and participants were asked for any questions regarding the way to complete the questionnaire. The purpose of the VMIQ-2 was not mentioned. The factorial structure of the VMIQ-2 questionnaire according to the different MI modalities was assessed by mean of confirmatory factor analysis, while construct validity study of the questionnaire provided Cronbach’s alpha coefficients superior to 0.90 in all MI modalities [37].

As instructed by the VMIQ-2 questionnaire, participants were not allowed to provide similar ratings to different modalities of a given MI item. The general VMIQ-2 score (i.e. including all subscores) as well as EVI and IVI subscores were collected. Kinesthetic MI was considered too complex for athletes naive to MI practice. Therefore, kinesthetic subscores were not considered. During the preliminary session, experimenters checked that all athletes were able to distinguish EVI from IVI without switching between the two perspectives. Two homemade 10 s breaststroke swimming video clips were individually presented at the beginning of the preliminary session, so as to illustrate IVI and EVI. The first video clip was filmed at the third-person, while the second one was filmed at the first-person using a waterproof video camera fixed on the swimmer’s head. The same athlete appeared in the two video clips. After experimenters asked to the participants whether they understood the distinction between IVI and EVI, they individually performed 10 s of backstroke swimming using EVI and IVI (one MI trial each). After each MI task, they were requested to orally describe the content of their MI.

**Figure 1:**
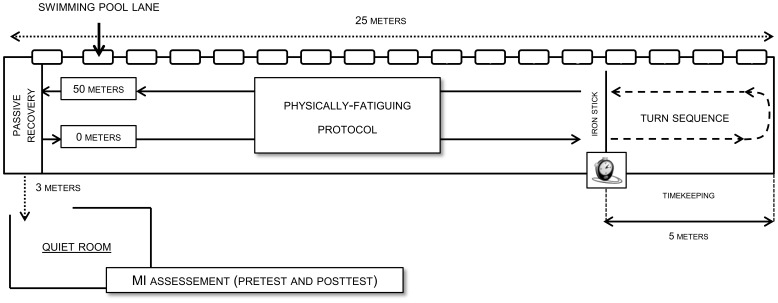
Experimental setting. MI  =  motor imagery.

Two repeated assessments of MI accuracy were then performed during the experimental session, which was scheduled one week after the preliminary session. The first was completed immediately after a 20 min non-fatiguing warm-up session (pretest) consisting in 10 minutes of freestyle swimming at 50–55% of the MAS. The second was performed after a 45 min physically-fatiguing freestyle swimming protocol (posttest). Participants repeatedly performed (i.e. using an interval-training design) a 50 m distance (Fig. 1) from 70% to 100% of their MAS. Passive recovery periods (i.e. 5 to 15 s periods where swimmers remained motionless at pool ending) were allocated throughout the fatiguing protocol, for a total amount of 15 minutes of passive recovery allocated along the course of the 45 min incremental exercise. All changes in swim pace were specifically instructed and controlled by the experimenter: times required to perform the 50 m matched the theoretical duration swimmers would achieve to perform the 50 m distance when swimming at the instructed percentages of their MAS (considering that the 10 m corresponding to turns should be systematically performed at 90% of the MAS).

### Experimental Settings

Both preliminary and experimental sessions were individually implemented for each participant and took place in a 25 m swimming pool. The ending portion of the pool corresponding to turn was materialized 5 m from the ending wall by iron sticks dived at the bottom of the pool, perpendicularly from the lane (Fig. 1). During both warm-up and physically-fatiguing protocol, swimmers were asked to perform the turn sequence, i.e. the portion of the pool ranging from the iron stick to the pool ending (forth and back1), at 90% of their MAS, independently from instructed changes in swim pace. MI accuracy assessments were completed after both pre- and posttest in a quiet room immediately close to the pool.

### Actual Turn Sequence Time Recording

During both warm-up and physically-fatiguing protocol, we recorded the duration of the turn sequences (PP times). Timekeeping was triggered when swimmer’s head passed above the iron stick (i.e. entered the pool portion ranging from the iron stick to the ending wall 5 m ahead, Fig. 1), and stopped when swimmer’s head reached the iron stick after the turn. A turn sequence therefore included wall approach, somersault turn and stroke recovery. Timekeeping was manually performed by a professional swimming coach who was not informed about the purpose of the study. Before each experimental session, he was requested to perform the timekeeping of four video motor sequences lasting from 5 to 15 s. Our purpose was to check the accuracy of the timer used along the course of the experiments (timer “Interval 2000” Nielsen Kellerman®). Intra-rater reliability did not elicit any significant difference between actual times and times recorded by the timekeeper, using paired comparisons (t = 0.30, p = 0.77, non significant mean of the absolute differences being 0.046 s).

### Control of Physical Fatigue

We recorded the electrocardiogram and expressed cardiac activity in the form of Instantaneous Heart Rate (IHR) before and after warm up, and at 15, 30 and 45 min during the physically-fatiguing protocol. We used a thoracic heart rate monitor (Polar® S810i) from a standing position. During MI assessments (pre- and posttest), HR was continuously recorded as a marker of energy expenditure. Participants also rated task difficulty after both warm-up and physically-fatiguing protocol, using the Rating Perceived Exertion scale [38] ranging from 6 (“extremely easy”) to 20 (“extremely difficult”).

### Pretest and Posttest MI Assessments

Pretest and posttest consisted in two blocks of 10 MI trials where participants mentally represented themselves executing the turn sequence at 90% of their MAS (i.e. the instructed swim pace to physically execute the turn sequence during both warm-up and physically fatiguing protocol). MI trials were performed from a standing up body position using IVI and EVI as presented randomly (10 MI trials in each modality). Pretest and posttest were supervised by the experimenter who provided verbal instructions regarding the MI task (“*Mentally represent yourself performing a freestyle swimming turn sequence at 90% of your MAS, starting once your head passes above the iron stick - thus entering the pool portion ranging from the iron stick to the ending wall 5* *m ahead - until your head reaches the iron stick back after the turn. You will be told before each trial whether to use EVI or IVI*.”). He was assisted by the professional swimming coach for timekeeping MI times. A 20 s period separated the two blocks of MI trials. No specific instructions were provided regarding whether participants should perform MI with their eyes closed or open. At the exception of one participant who opened his eyes during both pre- and posttest, all participants performed MI trials with closed eyes. During the posttest, we controlled that participants did not recover up to the maximal HR value they reached during the pretest.

Reliable MI assessment ideally requires combining neurophysiological and psychological methods [39]. Firstly, we collected MI times. Participants orally indicated when they mentally entered and exited the pool portion corresponding to the turn sequence (i.e. when their head reached the iron stick, forth and back). We investigated participant’s ability to achieve congruence between the temporal structure of imagined actions and the actual timing of motor performance (see data analysis section below for description of the statistical treatment) as an indicator of MI accuracy [22]. To avoid any interference with potentially deleterious effects of physical fatigue on PP times, PP times recorded during the warm-up were considered the reference for further comparisons with both pretest and posttest MI times.

**Figure 2:**
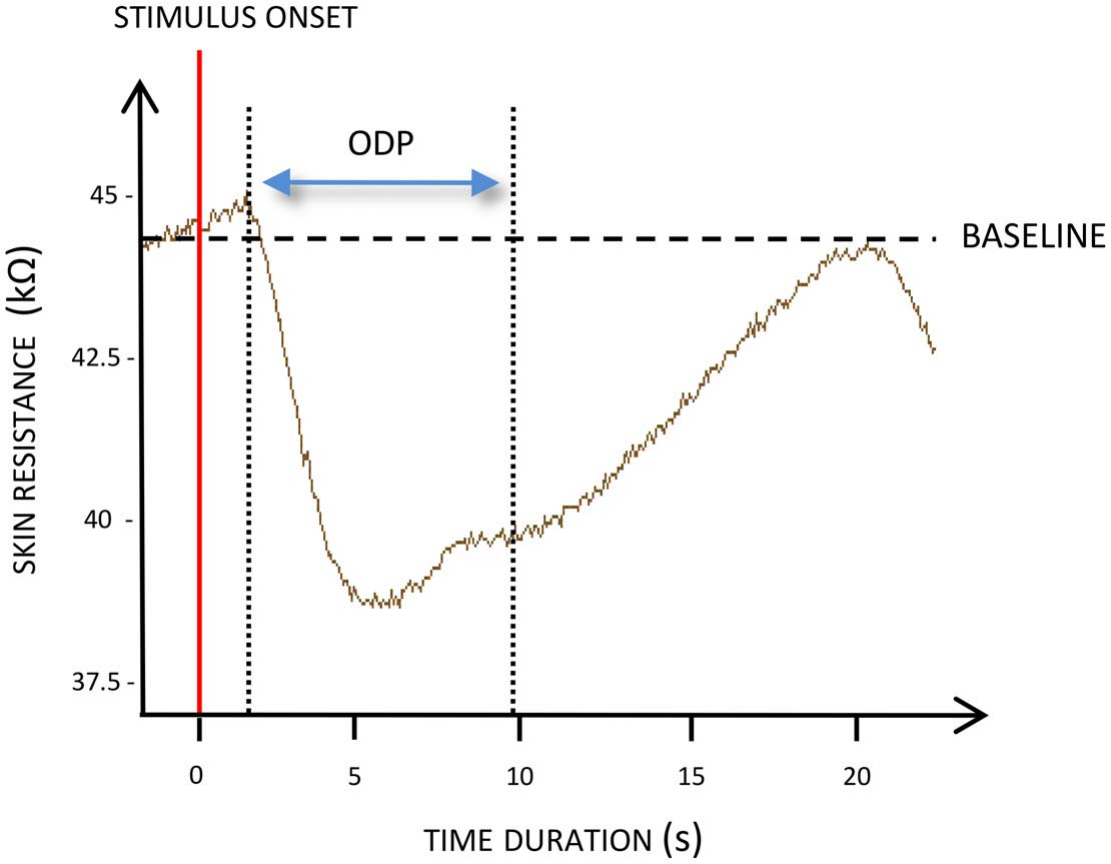
A representative example of OPD during MI (S07).

Autonomic Nervous System (ANS) responses are generally elicited as early as participants start to mentally represent themselves performing an action, hence providing a reliable index in the assessment of MI accuracy [39]. Specifically, we recorded skin resistance responses using two 30 mm^2^ unpolarizable Ag/AgCl electrodes (Clark Electromedical Instruments, Ref. E243) placed on the second phalanx of the second and third digits of the non-dominant hand, held by adhesive tape. Isotonic conductive paste was used to improve skin/electrode contact. Skin resistance was recorded with 5 µA current (current density = 10 µA/cm^2^). We specifically took the Ohmic Perturbation Duration (OPD) as dependent variable. OPD was measured from the sudden baseline drop elicited by MI, and the end of electrodermal response, i.e. when the slope resembled that observed before stimulation, with no micro fluctuation, while recovering basal level [40] (Fig. 2). OPD is directly elicited by orthosympathetic endings activity, innervating sweat glands, in response to MI. Further, OPD was demonstrated an objective neurophysiological correlate of MI vividness [5].

At the end of both pretest and posttest, participants rated their difficulty at performing MI on a 10-point Likert-type scale (10 corresponding to “maximal difficulty”). They gave a general rating based on all MI trials, then a specific rating for EVI and IVI trials.

### Data Analysis

As VMIQ-2 scores are discrete variables, MI profile (i.e. the individual MI perspective preference) was obtained by comparing IVI and EVI scores with Wilcoxon paired-test. We then used analyses of variance (ANOVAs) with repeated measures to compare pre- and posttest PP times. Likewise, we used repeated measures ANOVAs to compare IHR values before and after warm-up, as well as during the physically-fatiguing protocol. Post-hoc tests were performed with Bonferroni corrections.

ANOVAs with repeated measures further compared warm-up PP times, pretest MI times, and posttest MI times to assess temporal congruence between MI times in pretest and posttest and the timing of actual motor performance. Likewise, we compared OPD values and Likert ratings between pre- and posttest. We used paired t-tests with Bonferroni correction for post-hoc comparisons. We calculated the difference between posttest and pretest MI times and OPD, thus providing ΔMI times and ΔOPD values for each participant. Similarly, we obtained the difference between post- and pretest self-reports ratings (Likert self-reports, ΔLSR). Correlations between ΔMI times and ΔOPD on the one-hand, and ΔMI times and VMIQ-2 scores on the other, were computed to test whether the subjective experience of MI vividness was linked to the effect of physical fatigue on dependent variables. We also compared pre- and posttest self-report ratings using ANOVAs with repeated measures, and examined the correlation between ΔLSR and both ΔMI times and ΔOPD. We finally studied the selective effect of physical fatigue on EVI and IVI with a similar procedure. We used R freeware for all data and statistical computing with a type 1 error rate of α = 0.05 for statistical significance.

## Results

### VMIQ-2 Questionnaire

All participants reported that they did not switch from one perspective to another during MI and that they were able distinguishing IVI from EVI. Five participants exhibited higher IVI than EVI scores, while six had similar scores and one reported significantly higher EVI scores (Table 1). When pooled, all VMIQ-2 items provided a mean score of 2.18 (CI 95% = 0.28) on the 5-point scale (5 indicating “absence of mental image”). Respective mean EVI and IVI scores were 2.33 (CI 95% = 0.40) and 2.08 (CI 95% = 0.26). Thus, participants were able to form “*quite neat and vivid images*” to “*moderately neat and vivid images*” in both MI perspectives.

**Table 1:** Mean VMIQ-2 with EVI and IVI sub-scores (± standard errors).

VMIQ-2 General		EVI subscore	IVI subscore	Wilcoxon test	MI pofile
**S1**	2.00±0.13	2.25±0.22	1.58±0.26	W = 61.50, p = 0.05	IVI
**S2**	2.47±0.17	2.58±0.28	1.91±0.19	W = 65.00, p = 0.02	IVI
**S3**	1.52±0.09	1.08±0.08	2.08±0.08	W = 0.00, p<0.001	EVI
**S4**	3.00±0.20	3.16±0.24	2.75±0.40	W = 54.00, p = 0.21, NS	None
**S5**	1.72±0.12	1.41±0.14	2.16±0.23	W = 30.50, p = 0.50, NS	None
**S6**	2.97±0.17	3.00±0.29	2.91±0.33	W = 35.50, p = 0.84, NS	None
**S7**	1.94±0.14	1.75±0.50	2.33±0.28	W = 18.00, p = 0.07	None
**S8**	2.47±0.14	3.00±0.17	1.91±0.25	W = 72.50, p = 0.006	IVI
**S9**	1.77±0.13	2.08±0.22	1.91±0.22	W = 38.50, p = 0.62, NS	None
**S10**	1.97±0.14	3.00±0.25	1.33±0.14	W = 78.00, p = 0.002	IVI
**S11**	2.38±0.15	2.58±0.33	2.16±0.24	W = 51.50, p = 0.32, NS	None
**S12**	1.91±0.12	2.27±0.18	1.54±0.15	W = 40.50, p = 0.02	IVI

VMIQ-2 =  Vividness Movement and Imagery Questionnaire 2, MI  =  motor imagery, EVI  =  external visual imagery, IVI  =  internal visual imagery. NS: non significant.

### Control of the Physical Fatigue

**Figure 3:**
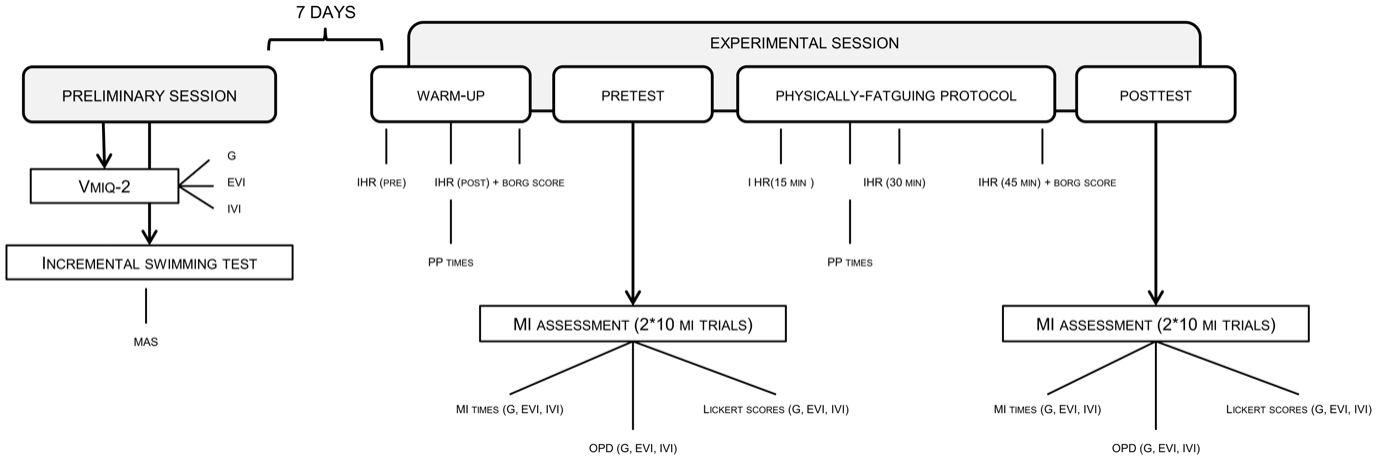
Experimental design. VMIQ-2 =  Vividness of Movement Imagery Questionnaire 2, G  =  general, EVI =  external visual imagery, IVI  =  internal visual imagery, MAS  =  maximal aerobic speed, IHR  =  instantaneous heart rate, PP  =  physical practice, MI  =  motor imagery, OPD  =  Ohmic Perturbation Duration.

Mean IHR rates increased from 86.50 bpm (IC 95%  = 7.83) before warm-up, to 125.75 bpm (IC 95% = 12.11) after (Fig. 3). Regular and progressive increase occurred during the fatiguing protocol to reach 194 bpm (IC 95%  = 8.73) at the end of the session. Obviously, ANOVA confirmed significant IHR increase from rest to warm-up, and from warm-up to the end of the fatiguing protocol (F = 59.7, p<0.001 and F = 39.6, p<0.001, respectively). As instructed, PP duration of the turn sequence did not differ between warm-up and fatiguing protocol sessions (F = 1.13, p = 0.30, NS; Fig. 3).

Finally, self-report ratings on the RPE Borg scale revealed that participants perceived the warm-up as being “very easy” (mean = 9.25, IC 95% = 0.76), while self-estimation ranged from “difficult” to “very difficult” (mean = 16.41, IC 95% = 0.74) after the physically-fatiguing protocol.

### Effect of Physical Fatigue on MI Ability

**Figure 4:**
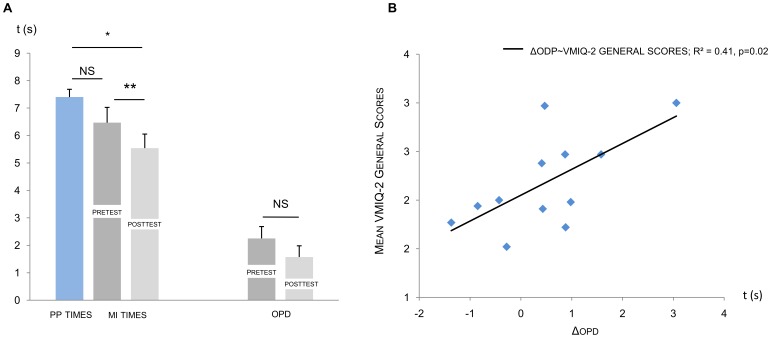
Mean imagined duration and ohmic perturbation duration (standard errors). PP  =  physical practice, MI  =  motor imagery, OPD  =  ohmic perturbation duration, *  =  p<0.05, **  =  p<0.01, NS  =  Non-Significant difference (p>0.05). D: Correlations between posttest minus pretest imagined times/ohmic perturbation durations and VMIQ-2 general scores. ΔOPD  =  posttest minus pretest ohmic perturbation durations, ΔMI times  =  posttest minus pretest motor imagery times.

ANOVA with repeated measures revealed the significant effect of repeated measurements between pretest MI times, posttest MI times, and PP times (F = 4.97, p = 0.01, η^2^ = 0.31). Post-hoc tests with Bonferroni correction indicated that while there was no difference between PP times (mean = 7.49, s.e. = 0.24) and MI times (mean = 6.47; s.e. = 0.55) during the pretest (p = 0.22, NS), MI duration was significantly shortened during the posttest (p = 0.01; Fig. 4A). Furthermore, as compared to pretest MI times, posttest MI times (mean = 5.54; s.e. = 0.51) were significantly shortened (p<0.01; Fig. 4A). Conversely, the effect of physical fatigue on OPD was inconclusive (Table 2).

**Table 2:** Statistical analyses performed on general scores.

Repeated measures ANOVA		Post-hoc comparisons with paired t-tests		
		PP vs. pretest MI times	PP vs. posttest MItimes	Pretest vs. posttest MI times
MI and PP Times	F = 4.97, p = 0.01	t = 1.27, p = 0.22, NS	t = 2.82, p = 0.01	t = 3.43, p<0.001
OPD	F = 2.36, p = 0.18, NS			
LSR	F = 2.2, p = 0.17, NS			

MI = Motor Imagery, PP =  Physical Practice, OPD =  Ohmic Perturbation Duration, LSR =  Likert Self-Reports. NS: non significant.

General VMIQ-2 scores were significantly correlated to ΔOPD (R^2^ = 0.41, p = 0.02, Fig. 4B), but not to ΔMI durations (R^2^ = 0.07, p = 0.39). Interestingly, participant’s self-reports on the Likert scale did not differ between pre- and posttest sessions (F = 2.2, p = 0.17, η^2^ = 0.16), and no significant correlation was found between general ΔLSR and both ΔMI duration (R^2^ = 0.01, p = 0.76) and ΔOPD (R^2^ = 0.05, p = 0.48).

### Selective Effect of Physical Fatigue on IVI and EVI

**Figure 5:**
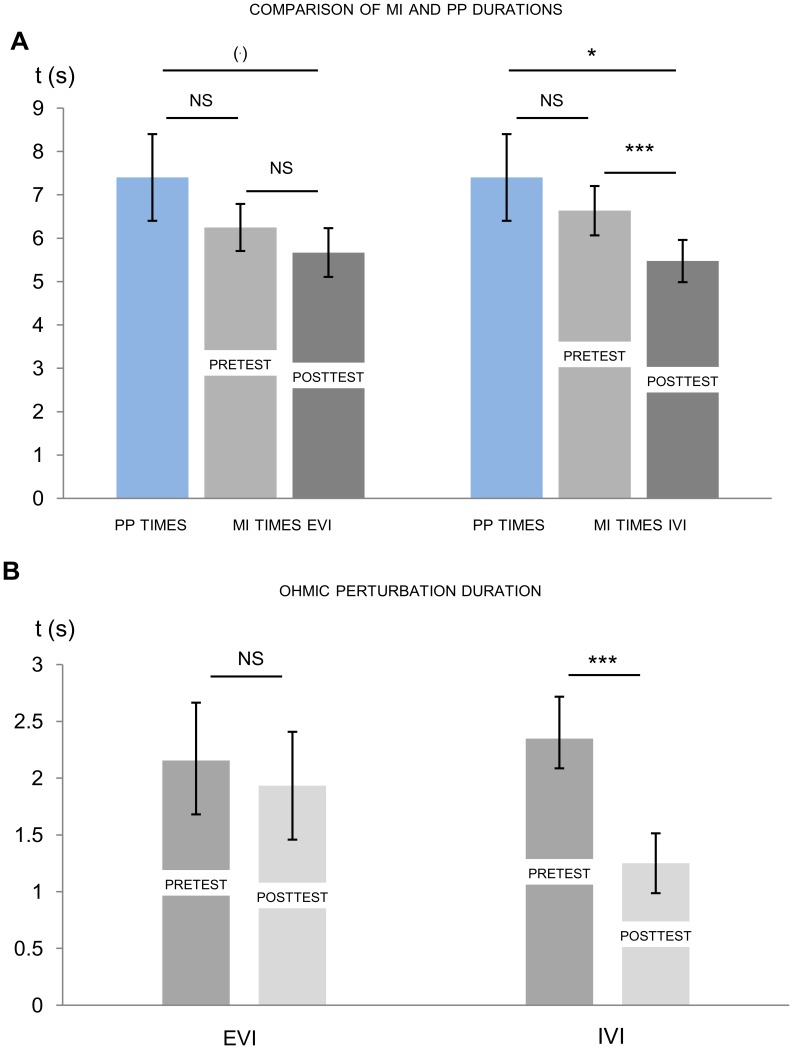
E: Mean (standard error) physical practice (PP) times and imagined times in pretest and posttest. MI  =  motor imagery, EVI  =  external visual imagery, IVI  =  internal visual imagery, (.)  =  trend to significance (0.02<p<0.05), NS = p>0.05, * = p<0.05, *** = p<0.001, NS  =  Non-Significant difference (p>0.05). F: Mean (standard error) ohmic perturbation durations (OPD) before (pretest) and after (posttest) physical fatigue. EVI  =  external visual imagery, IVI  =  internal visual imagery.

ANOVAs performed on MI and PP times reached significance in both EVI (F = 3.95, p = 0.03, η^2^ = 0.26) and IVI (F = 5.68, p = 0.01, η^2^ = 0.34). During EVI and IVI, PP and pretest MI times were similar (Table 3). During EVI, the difference between PP duration and posttest MI duration did not reach significance (p = 0.03) when applying Bonferroni correction (p_Bonferroni(k = 3)_ = 0.02), while significantly shorter posttest MI times were recorded during IVI as compared to PP duration (p = 0.01; Fig. 5A). When comparing pretest to posttest MI duration (i.e. the effect of the physically-fatiguing protocol), data revealed shortened duration during IVI posttest (p<0.001), whereas there was no effect of physical fatigue on MI duration during EVI (p = 0.18; Fig. 5A).

**Table 3:** Analysis of external and internal visual imagery subscores, separately.

Repeated measures ANOVAExternal Visual Imagery		Post-hoc comparisons with paired t-tests			
		PP vs. pretest MI times	PP vs. posttest MI times	Pretest vs. posttest MI times	
MI and PP Times	F = 3.95, p = 0.03	t = 1.58, p = 0.14, NS	t = 2.48, p = 0.03	t = 1.42, p = 0.18, NS	
OPD	F = 0.55, p = 0.47, NS				
LSR	F = 0.16, p = 0.68, NS				
**Repeated measures ANOVA** **Internal Visual Imagery**		**Post-hoc comparisons with paired t-tests**			
		**PP vs. pretest MI times**	**PP vs. posttest MI** **times**		**Pretest vs. posttest MI times**
MI and PP Times	F = 5.68, p = 0.01	t = 1.03, p = 0.32, NS	t = 3.08, p = 0.01		t = 5.32, p<0.001
OPD	F = 27.7, p<0.001				
LSR	F = 3.39, p = 0.09 NS				

MI = Motor Imagery, PP =  Physical Practice, OPD =  Ohmic Perturbation Duration, LSR =  Likert Self-Reports, Δ  =  Delta posttest minus pretest values. NS: non significant.

Likewise, OPD remained unchanged between pre- and posttest sessions during EVI (F = 0.55, p = 0.47, NS, η^2^ = 0.05), whereas we recorded significantly shorter OPD during IVI posttest (F = 27.7, p<0.001, η^2^ = 0.71, Fig. 5B).

ANOVA comparisons of pretest and posttest self-report ratings did not reveal any significant difference during EVI (F = 0.16, p = 0.68, NS, η^2^ = 0.01), while ratings during the posttest were slightly higher without reaching significance during IVI (F = 3.39, p = 0.09, η^2^ = 0.24).

**Figure 6:**
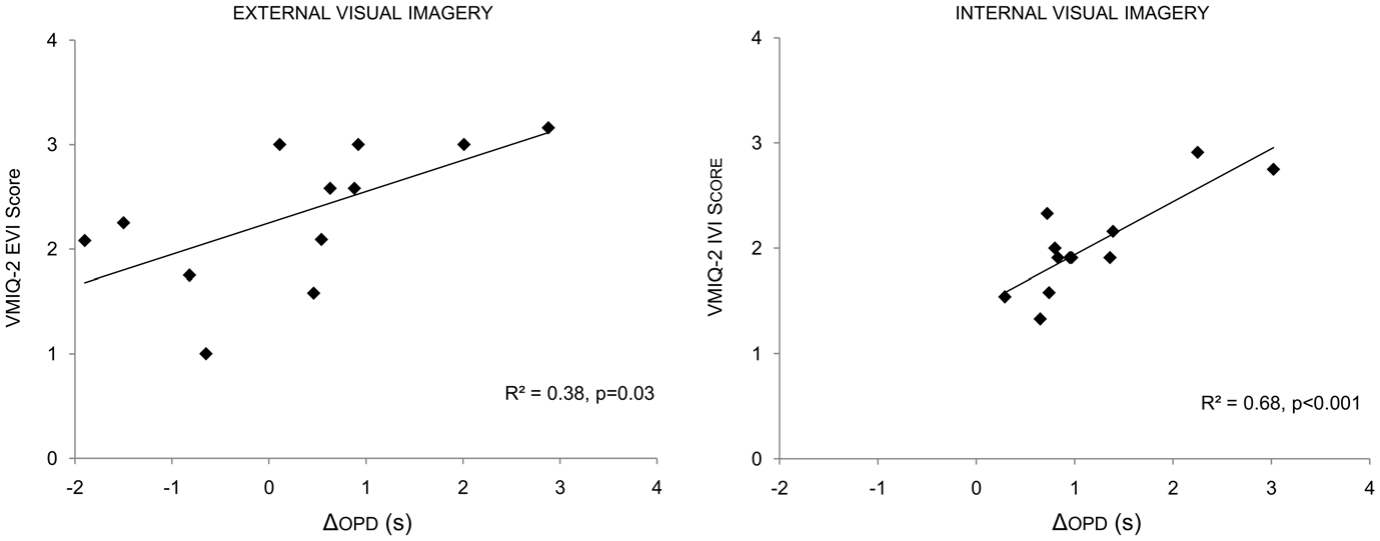
Correlation between VMIQ-2 subscores (y axis) and pretest versus posttest difference in ohmic perturbation duration. VMIQ-2 =  Vividness of Movement Imagery Questionnaire 2, ΔOPD  =  posttest minus pretest ohmic perturbation durations, EVI  =  external visual imagery, IVI  =  internal visual imagery.

ΔLSR during EVI did not correlate to either ΔMI duration or ΔOPD (R^2^ = 0.02, p = 0.64; R^2^ = 0.06, p = 0.44). Similarly, ΔLSR during IVI did not correlate to either ΔMI duration or ΔOPD (Table 4). Conversely, VMIQ-2 scores and ΔOPD exhibited significant correlations in both EVI and IVI (R^2^ = 0.38, p = 0.03; R^2^ = 0.68, p<0.001, respectively; Fig. 6) while VMIQ-2 subscores and ΔMI times were not correlated in both MI modalities (Table 4).

**Table 4:** Correlations carried on general and specific internal and external motor imagery subscores.

Correlations	General Scores	IVI Scores	EVI Scores
**VMIQ-2∼ΔMI Times**	R^2^ = 0.07, p = 0.39, NS	R^2^ = 0.16, p = 0.20, NS	R^2^ = 0.007, p = 0.78, NS
**VMIQ-2∼ΔOPD**	R^2^ = 0.41, p = 0.02	R^2^ = 0.38, p = 0.03	R^2^ = 0.68, p<0.001
**ΔLSR∼ΔMI Times**	R^2^ = 0.01, p = 0.76, NS	R^2^ = 0.02, p = 0.64, NS	R^2^ = 0.006, p = 0.80, NS
**ΔLSR∼ΔOPD**	R^2^ = 0.01, p = 0.48, NS	R^2^ = 0.06, p = 0.44, NS	R^2^ = 0.01, p = 0.71, NS

VMIQ-2 =  Visual Movement Imagery Questionnaire 2, IVI =  Internal Visual Imagery, External Visual Imagery, MI = Motor Imagery, PP =  Physical Practice, OPD =  Ohmic Perturbation Duration, LSR =  Likert Self-Reports, Δ  =  Delta posttest minus pretest values. NS: non significant.

## Discussion

The main purpose of this study was to examine the effect of physical fatigue occurring during an intense sport training session on MI ability. We postulated that MI quality might be affected by physical fatigue from which athletes do not recover rapidly. We further investigated the selective effect of physical fatigue on IVI and EVI, as we considered that physical fatigue might differently affect MI accuracy depending on different individual MI abilities in the two modalities.

In the present study, MI accuracy was assessed before and after an intense physically-fatiguing training session. We used a well-learned routine of the sport practiced to assess MI ability *i.e.* participants had to mentally perform a swim turn from the last 5 m before reaching the wall to the first 5 m following swim turn. Our main objective was to avoid an experimental design decoupled from the context of actual training practice. Therefore, we selected a goal-directed movement, highly automated after physical rehearsal. We assessed MI accuracy using a validated set of psychometric, behavioral and physiological tests [39].

As expected, warm-up did not elicit physical fatigue based on moderate IHR increase and from self-reports, *i.e.* participants estimated that the warm-up was “very easy”. In other words, the physical practice performed during warm-up could not be considered a confounding factor. Conversely, the 45 min exercise protocol elicited intense physical fatigue. Mean IHR reached 194 bpm, while participants perceived the session from “difficult” to “very difficult”. Despite this, during both warm up and the physically-fatiguing protocol, swimmers sustained the expected regular swim speed during turn sequences, as requested. Therefore, differences between pre- and posttest sessions may not account for differences in task practice. We finally excluded a potential age-related effect. Albeit possible, several researchers provided evidence that adolescents are able to imagine in real-time and that MI ability is fully developed and comparable to that of an adult since the age of 14, including the use of different imagery types [41]–[45].

Interestingly, data showed a general effect of physical fatigue on MI duration. Shorter MI duration was recorded during the posttest, hence suggesting that participants encountered greater difficulty to achieve the temporal congruence between MI and PP under fatigue. By contrast, similar neurophysiological correlates of MI were observed before and after fatigue. Taken together, these results indicate that physical fatigue might primarily affect the temporal organization of MI, while MI vividness would not be altered. Looking at the selective effect of physical fatigue on IVI and EVI however revealed that both MI duration and vividness were substantially affected when athletes used IVI, whereas there was no actual influence of fatigue when performing EVI. These data suggest that the effect of physical fatigue on MI ability is dependent on imagery content, and might therefore not be due to a general perception of muscular fatigue, as earlier postulated by Demougeot and Papaxanthis [36]. These results further promote the importance of taking the MI perspective into account when studying this mental process.

Participants reported similar difficulty when performing MI during pre- and posttest sessions. This result was also observed when considering specific EVI and IVI ratings on the Likert scale. Further, ΔSRs were not correlated to ΔMI duration or ΔOPD, thus indicating that the subjective experience of MI practice remained unchanged in spite of altered MI accuracy during IVI. Nonetheless, participants tended to report more difficulty to perform IVI after physical fatigue, while they did not report any trouble when using EVI. Therefore, whether participants consciously experienced the effect of physical fatigue on their ability to form accurate mental images remains questionable.

The VMIQ-2 scores revealed that 5 participants out of 12 presented significantly higher IVI than EVI scores, while only one reported higher EVI ratings. In participants with a marked MI profile (i.e. significantly different EVI and IVI scores at the VMIQ-2 questionnaire), the reasonwhy IVI modality outperformed EVI might be explained by the fact that swimmers do not gain frequently access to an external representation of their swim, i.e. imagining themselves swimming from an external viewpoint. The VMIQ-2 general score was not correlated to ΔMI duration. Conversely, ΔOPD significantly co-varied with VMIQ-2 general score in spite non-significant general effect of physical fatigue on ODP. Interestingly, these data indicate that swimmers who subjectively perceived vivid visual images were the less strongly impacted by physical fatigue with regards to MI vividness, as estimated via an objective neurophysiological correlate. EVI, IVI VMIQ-2 scores and ΔOPD also presented significant correlations.

We early postulated that difference in MI expertise between IVI and EVI might explain the selective effect of physical fatigue on IVI and EVI. As swimmers were likely to better perform IVI than EVI, IVI could potentially be more affected by physical fatigue while EVI accuracy would remain poor. Several considerations however disregard this hypothesis. Firstly, even though VMIQ-2 results highlighted a preference for IVI, 6 swimmers out of 12 obtained comparable IVI and EVI scores. Secondly, participants achieved temporal congruence between actual practice and MI in both imagery perspectives during the pretest, hence suggesting comparable MI ability between the two modalities. Finally, as previously mentioned, VMIQ-2 scores and ΔOPD were negatively correlated, hence indicating that the better swimmers performed during EVI and IVI, the less physical fatigue impacted MI vividness. These findings therefore challenge our initial hypothesis. As participants achieved the temporal congruence between MI and PP during the pretest, the observed effect of physical fatigue when performing IVI might account for central processes affecting the internal representation of the motor sequence. This postulate is congruent with previous findings. Demougeot and Papaxanthis [36] argued that the effect of fatigue on MI accuracy may result from altered ability of the central nervous system to predict the sensorial consequences of subsequent actions, *i.e.* forward models [46]. Forward models integrate the actual state of the motor system and contribute to predict both mental and actual motor executions, and are *“not fixed entities but (…) updated through experience”* [46]. Here, we assume that physical fatigue elicited by prolonged and intense exercise might have affected the way in which participants experienced the turn sequence, probably affecting its internal representation within long-term memory. Indeed, MI is supported by motor representations recalled within working memory [47], [48]. Decreased MI accuracy may thus account for the effect of physical fatigue on the subjective experience of PP. This point is further congruent with self-reports spontaneously made by swimmers after the experiment. They explained that they tended to omit some portions of the turn sequence while performing IVI (e.g. the wall approach). Such process might further explain the observed modality-dependent effect of physical fatigue, as EVI is namely based on “external” representation of actions, therefore less tightly related to individual motor experience than IVI. This assumption is congruent with recent neuroimaging findings supporting the embodied nature of IVI as compared to EVI [30].

At first glance, present data seem to challenge previous results by Guillot et al. [35], who initially observed that both MI duration and ANS responses recorded during MI were not strongly affected by local muscular fatigue elicited by repetitive squat-jumps. Looking at the experimental design however reveals that the duration of the fatiguing session was very short (ranging between 1 and 2 min), and included a limited number of repetitions. We therefore postulate that muscle fatigue might not have altered the internal motor representation of the squat-jump movement, thus preserving MI accuracy. Guillot et al. [35] further mentioned in their conclusions that fatigue elicited by more prolonged physical activity might have more deleterious effects on MI accuracy. Here, we provide congruent data to their hypothesis, as peripheral fatigue elicited by prolonged incremental exercise affected both IVI accuracy and timing. Accordingly, we suggest that the co-dependent interaction between peripheral fatigue and MI ability might depend on the nature of the fatigue elicited. Also, we postulate that physical fatigue may differently affect MI vividness depending on the individual MI ability, as participants who reported forming vivid images (using the VMIQ-2) were less strongly impacted by physical fatigue. Good imagers might run a stable internal representation of the movement during MI, mediated by specific neural processes as compared to poor imagers [49], and therefore less likely to be updated due to temporary feedback originating from actual practice.

Based on previous data and present results, we may conclude that physical fatigue is likely, albeit not systematically, to affect MI timing and vividness. More data are needed to further delineate how peripheral changes may affect motor-related mental processes.

While local muscle fatigue may have no detrimental influence on MI timing, present data show that physical fatigue occurring during an intense sport session altered MI ability when MI was internally performed. These findings further support that considering the actual state of fatigue should be integrated in future models examining practical applications of MI. As real-time updating of motor representations derived from sensory feedback are likely to affect MI accuracy, then physically-fatiguing protocols conducted until exhaustion (i.e. eliciting degradation of the actual motor performance) may induce differential changes in MI accuracy, for instance increased MI times in the case of slower motor performance.
